# Enhancing Pain Management for Open Inguinal Hernia Repair With a Novel Long-Acting Bupivacaine Drug Device (Xaracoll®) in Multimodal Pain Control: A Quality Improvement Initiative to Reduce Post-Operative Opioid Prescribing

**DOI:** 10.7759/cureus.53648

**Published:** 2024-02-05

**Authors:** Julia Scott, Baraa Souman, Muhammad Darwish, Mark Farro, Charles Chesnut

**Affiliations:** 1 Department of Medicine, Touro University Nevada College of Osteopathic Medicine, Henderson, USA; 2 Department of General Surgery, Valley Health System, Las Vegas, USA; 3 Department of Pharmacy, Spring Valley Hospital, Las Vegas, USA

**Keywords:** quality improvement, local anesthetic, postoperative opioid consumption, opioid-sparing analgesia, open inguinal hernia repair, multimodal pain control

## Abstract

Background

Xaracoll® is a Food and Drug Administration (FDA) approved Type 1 Bovine collagen-based bupivacaine hydrochloride (HCl) implant developed to provide postoperative pain management for up to 24 hours after open inguinal hernia repair in adults. This retrospective review examined the efficacy of Xaracoll® in the management of postoperative pain compared to injectable Bupivacaine.

Methods

This retrospective study examines 54 patients who underwent unilateral open inguinal hernia repair by a single surgeon over three years. The control group consisted of 36 patients who received injectable Bupivacaine as the local anesthetic. Eighteen patients received the Xaracoll® drug device intra-operatively following the FDA-approved manufacturer’s guidelines. Intra-operative analgesics administered and quantified by oral morphine equivalents (OME), opioid administration for pain control postoperatively, opioid prescriptions upon discharge, postoperative pain scores, and turnaround time (TAT) were compared.

Results

The use of Xaracoll® in inguinal hernia repair is associated with a decrease in the rate of opioid administration in the post-anesthesia care unit (PACU) (22.2% vs. 52.8%; p = 0.043). In addition, patients requiring opioids in the outpatient setting needed significantly less OME in the Xaracoll® group compared to the control group (52.50 vs. 136.15; p < .001).

Conclusion

This study demonstrates compelling evidence that Xaracoll® is a useful analgesia adjuvant for inguinal hernia repair, significantly reducing the need for opioids in the PACU and decreasing doses of opioid medications upon discharge. Xaracoll® is effective in minimizing postoperative pain and opioid medication dosages upon discharge as part of a multimodal approach to pain and improving patient experience. Further research is warranted to evaluate Xaracoll®’s role in pain control in the PACU and on discharge.

## Introduction

Opioids are an essential category of analgesics and play an important role in controlling postoperative pain. However, opioid-involved deaths increased by 38% in the United States, and prescription opioid-involved deaths increased by 17% from 2019 to 2020 [1]. The increasing rates of opioid misuse, addiction, and overdose prompt an important discussion concerning the practices of opioid prescription. Surgery is an important risk factor in the development of opioid misuse disorder, and one study found that up to 6% of patients undergoing major and minor surgeries continued to use opioids 90 days postoperatively [2,3]. Surgeons can help mitigate the opioid crisis and aid in safeguarding our communities by reducing inessential dosages of opioids provided postoperatively and by providing additional analgesia modalities to replace and reduce the need for opioids in postoperative pain control.

It is important to note that opioids given postoperatively are often over-prescribed and the cycle of over-prescription and improper disposal results in a large quantity of opioids being available for misuse [4,5]. In a study assessing the variation and excessive dosage of opioid prescriptions for common general surgical procedures, including inguinal hernia repair, Hill et al. found the mean number of opioid pills prescribed on discharge after open inguinal hernia repair was 33 [5]. In the same study, patients undergoing various general surgery procedures reported that over 70% of prescribed opioids were never taken, and only 9% of patients disposed of their surplus opioids according to FDA recommendations [5].

A retrospective study of over 59,000 opioid-naive patients who underwent inguinal hernia repair found that 1.5% developed new persistent opioid use postoperatively [6]. While patient and community-wide education on proper opioid disposal may contribute to reducing the number of prescription opioids available for misuse in the community, additional strategies are needed to combat the multifaceted problem of the opioid crisis.

Integration of enhanced recovery after surgery (ERAS) pathways with a multimodal, non-opioid pain management approach should be strongly considered as an alternative to opioids for the management of postoperative pain in patients undergoing open inguinal hernia repair [7,8]. Local anesthesia at the conclusion of the procedure could be a component of this pathway, along with non-opioid analgesics. Inguinal hernia repair is often an outpatient procedure, and a study performed by Mylonas et al. reports that 110 out of 185 patients undergoing elective open inguinal hernia repair reported no opioid use postoperatively, even though they were given a prescription [9]. Those who did report opioid use postoperatively reported that they required less than five tablets [9]. Millard et al. also found that little to no opioid use was required of their patients after inguinal hernia repair [10]. These findings suggest that postoperative pain after inguinal hernia repair can be effectively controlled with non-opioid analgesics. Based on the results of a study by Knight et al., this conclusion can be made for both open and minimally invasive inguinal hernia repairs [11]. We suspect that the use of a long-acting local anesthetic implant, Xaracoll®, may further contribute to the reduction of opioid requirements for open inguinal hernia repairs as this device can provide local pain control for up to 24 hours post-operatively.

Local anesthetics should be considered as part of a multimodal pain management approach for inguinal hernia repairs. A study performed by Sandhu et al. showed that intermediate-acting local anesthetics such as Bupivacaine have been shown to provide a significant reduction in postoperative pain in patients undergoing truncal incisions [12]. However, injection of local Bupivacaine may only last up to 4 hours postoperatively while a long-acting implant such as Xaracoll® can last up to 24 hours postoperatively. Especially in the setting of outpatient surgery, this leaves patients with continued postoperative pain upon discharge home that has often been controlled with opioid medications. The use of a long-acting implant for local anesthesia may be able to provide prolonged postoperative pain control and reduce or eliminate the use of opioids for postoperative pain management after open inguinal hernia repairs [13].

We hypothesize that Xaracoll® may significantly decrease post-operative pain scores after open inguinal hernia repairs, decrease the rate of opioid prescriptions upon discharge, and decrease the post-operative time-to-discharge. Xaracoll® is a Type 1 bovine collagen-based long-acting bupivacaine-HCl implant that is FDA-approved for adults for placement following open inguinal hernia repair to provide analgesia for up to 24 hours postoperatively. This implant is absorbable and releases the active ingredient, bupivacaine, through diffusion into surrounding tissues, blocking the generation and conduction of nerve impulses to decrease the sensation of pain [14]. The released bupivacaine is absorbed systemically, metabolized primarily by the liver, and renally excreted [14].

## Materials and methods

A retrospective analysis of 54 patients over 18 years of age who underwent unilateral open inguinal hernia repair by a single surgeon over a period of two years (August 2021 - November 2022) was performed comparing Xaracoll® 300 mg to Bupivacaine-epinephrine (0.5% 150mg) 30mL after introduction of Xaracoll® to the facility in November 2021. All patients underwent elective repair of a non-recurrent unilateral inguinal hernia. A control group of 36 patients who had undergone open inguinal hernia repair prior to the introduction of Xaracoll® at the facility, with injectable Bupivacaine as the local anesthetic used, was compared to a study group of 18 patients who received the Xaracoll® drug device intraoperatively following the FDA approved manufacturer’s guidelines. Data collection was performed by the department of pharmacy for quality improvement purposes, de-identified and provided to our team. Due to less than minimal risk to study participants, this study was given an exemption by the institutional review board at Touro University Nevada.

Parameters evaluated included intra-operative oral morphine equivalents (OME) of opioids administered, pain scores in the post-anesthesia care unit (PACU), rate and OME of opioids administrated in the PACU, PACU turnaround time (time from arrival in the PACU to discharge), and rate and OME of prescribed opioid use after discharge. If patients were prescribed opioids after discharge, they were prescribed oxycodone 5mg or oxycodone-acetaminophen 5-325mg. Pain scores were based on the pain numeric rating scale (NRS), where 0 means “no pain” and 10 means “the worst possible pain”. Pain scores were obtained once, just prior to discharge from the PACU. Nonparametric univariate analysis was performed using the Mann-Whitney U and Fisher’s exact test for qualitative data. A p-value less than 0.05 was considered statistically significant. Statistical analysis was performed using JASP Team (2023) (JASP, Version 0.17.2, Computer software) [15]. Figures were produced using ggplot2 package (v3.4.2; Wickham, 2016) [16].

## Results

A total of 36 (66.7%) patients received Bupivacaine intraoperatively as a local analgesic and 18 (33.3%) patients received Xaracoll® intraoperatively. Mean pain scores in the PACU were higher in the Bupivacaine group compared to the Xaracoll® group (2.99 vs. 1.58) but did not reach statistical significance (p = 0.14). Nineteen (52.8%) patients in the Bupivacaine group required opioid administration in the PACU compared to four (22.2%) patients in the Xaracoll® group (p = 0.043) (Table 1), a 57.9% relative risk reduction in the Xaracoll® group. There was no statistically significant difference in the OME of opioids administered in the PACU between the two groups (p = 0.22) (Table 1). PACU turnaround time trended towards a quicker turnaround in the Xaracoll® group (84.9 vs 96.8 minutes) but did not reach statistical significance (p = 0.48).

**Table 1:** Summary of univariate analysis. Data is represented in mean ± standard deviation, number of patients, and percentage. A P-value of < 0.05 is considered significant. OME = oral morphine equivalents, PACU = post-anesthesia care unit, OR = odds ratio, TAT = turnaround time.

	Anesthetic			
	Bupivacaine (n=36)	Xaracoll® (n=18)	Statistical Parameter	p-value
Intraoperative OME	M = 16.61, SD = 11.81	M = 12.83, SD = 5.23	Mann-Whitney U. W = 391.0	p = 0.19
PACU opioids				
No	17 (47.2%)	14 (77.8%)	Fisher’s exact test. OR = 0.26 (95% CI: 0.07, 0.93)	p = 0.043
Yes	19 (52.8%)	4 (22.2%)		
PACU pain score	M = 2.99, SD = 3.51	M = 1.58, SD = 3.07	Mann-Whitney U. W = 394.0	p = 0.14
PACU OME	M = 15.46, SD = 15.12 (n = 19)	M = 16.88, SD = 6.25 (n = 4)	Mann-Whitney U. W = 22.50	p = 0.22
PACU TAT	M = 96.8, SD = 45.52	M = 84.9, SD = 17.70	Mann-Whitney U. W = 363.0	p = 0.48
Discharge opioids				
No	23 (63.9%)	10 (55.6%)	Fisher’s exact test. OR = 1.42 (95% CI: 0.45, 4.48)	p = 0.57
Yes	13 (36.1%)	8 (44.4%)		
Discharge OME	M = 136.15, SD = 41.30 (n = 13)	M = 52.50, SD = 26.59 (n = 8)	Mann-Whitney U. W = 100.0	p < .001

Upon discharge, 13 (36.1%) patients required opioids in the Bupivacaine group for pain management compared to eight (44.4%) in the Xaracoll® group (p = 4.48) (Table 1). However, there was a significant difference in the overall opioid amount used in the Xaracoll® group (52.50mg) compared to patients that received Bupivacaine (136.15, p < .001) (Table 1).

## Discussion

This study shows compelling evidence that Xaracoll® is a useful anesthesia adjuvant within a multi-modal pain management approach that can contribute to the reduction of the rate of opioid administration in the PACU and the mean OME of prescribed opioids in the outpatient setting. Xaracoll® utilization results in a relative risk reduction of 57.9% in the need for opioids for pain management in the PACU. These findings provide exciting evidence that Xaracoll® should be used as a component of multimodal pain control after unilateral inguinal hernia repair [13]. Given the increase in opioid-related deaths in the United States, as well as the risk for development of new persistent opioid use disorder, these results provide promising advances in non-opioid management of postoperative pain with Xaracoll® [1, 6].

Despite a similar mean OME of opioids administered in the PACU and no statistically significant difference in mean pain scores between the two groups, fewer patients required opioids in the Xaracoll® group. The lack of statistical difference in mean pain scores can likely be attributed to the study's small sample size given that, on average, patients receiving Xaracoll® had half the pain scores of those observed in the Bupivacaine group. These data provide compelling evidence that Xaracoll® is a useful tool in multimodal pain management to control postoperative pain and reduce opioid use postoperatively [13].

Another interesting finding was the reduction in the average OME required by patients needing opioids after discharge in the Xaracoll® group. This decrease is likely due to lower pain severity in the Xaracoll® group despite a similar outpatient opioid prescription rate compared to the Bupivacaine group. These findings suggest that Xaracoll® provided adequate pain control to our patients after discharge from the PACU despite the lower doses of opioids prescribed. Because surgery is a risk factor for the development of opioid use disorder, the reduction in opioid dosages found with Xaracoll® use may be important in limiting opioid exposure and decreasing rates of misuse postoperatively [3].

Our results for PACU turnaround time and postoperative pain scores also showed a reduction in the Xaracoll® group. These results provide insight that Xaracoll® may contribute to reduced PACU turnaround time and improved postoperative pain scores, and we plan to continue to investigate these variables in further studies.

For many procedures, including total knee arthroplasty, shoulder surgery, foot and ankle surgery, and colorectal surgery, regional anesthesia provides superior pain control to opioids [7]. While the comparison of pain control between Xaracoll® and opioids was not the goal of this study, this is an important topic to consider for future studies. In addition, further expansion of FDA approval of Xaracoll® may find that it can provide adequate or even superior pain control for other procedures in addition to open inguinal hernia repair.

We plan to continue our work to analyze the role of Xaracoll® in postoperative pain control. The trends of reduced PACU turnaround time (TAT) and PACU pain scores are very promising but were limited by our sample size and method of data collection. Demonstration of a consistent decrease in opioid pain medication requirements in PACU and discharge, combined with the other downstream effects of reduced TAT and pain scores in PACU, could shift the paradigm of postoperative pain management [13]. Furthermore, any expansion in the FDA approval for the utilization of Xaracoll® in other open surgery could expand this effect by several orders of magnitude, resulting in more opportunities for opioid reduction.

This study has several limitations, including the retrospective design. Selection bias must be considered, as the patients were not randomly assigned to the Bupivacaine group or Xaracoll® group, but rather were assigned based on timing before versus after the introduction of Xaracoll® to the facility. Because IRB approval was waived for this study, patient demographics were unable to be assessed in this study. In addition, the anesthesiologists assigned to the cases and their differing practice methods must be considered as a limitation of the study. There was no standardization of intraoperative pain management as administered by the anesthesiologists, and this influences the intra-operative OME administered. Likewise, the multimodal postoperative pain regimen was not standardized, further limiting the study. Finally, as this study was conducted remotely from the procedures, no follow-up was done to assess how many opioids prescribed were taken after discharge.

Further prospective studies with randomized control trial design need to be conducted to further investigate the role of Xaracoll® in postoperative pain control and reduction of post-operative opioid prescribing. These studies need to include controlled multimodal pre-operative and post-operative pain regimens, consistent intra-operative anesthesia, randomization of patient groups, and patient follow-up to assess the use of opioids after discharge.

## Conclusions

This study shows evidence that the use of Xaracoll® is associated with a reduction in the need for postoperative opioid administration and lower OME requirements on discharge after open unilateral inguinal hernia repair. These findings suggest that Xaracoll® is an effective alternative to traditional opioid analgesics for pain management in patients undergoing inguinal hernia repair, as well as an important component of multimodal pain control. We recommend that Xaracoll®, and the class of long-acting local anesthetics, be included as a standard component of all multimodal pain control strategies for indicated operations.
